# ‘Measuring’ Collective Trauma: a Quantum Social Science Approach

**DOI:** 10.1007/s12124-022-09696-2

**Published:** 2022-04-30

**Authors:** Kazuma Matoba

**Affiliations:** Department of Politics, Philosophy and Economics, Witten/Herdecke University, Witten/Germany, Niederhofenerstr. 4, D-44263 Dortmund, Germany

**Keywords:** Collective and intergenerational trauma, Quantum social science, Intra-action, Witnessing, Transparent communication

## Abstract

In the twenty-first century the world faces the stark reality that’s far from any visions of an ideal world, accompanied by an epidemic of social inequality and global injustice. Many social and global issues such as the refugee crisis, climate injustice, racism, whitism, and terrorism are rooted in serious, untreated historical traumata. These traumata have been experienced by one or more members of a family, group, or community, and may have been passed down from one generation to the next through epigenetic factors. Phenomena of collective trauma can be described more understandably through its interpretation by the quantum social science of Wendt (2016). This interpretation provides a social pathology that offers methodological recommendations (methods of treatment) for social therapy. One potential example is the collective trauma integration process (CTIP) developed by Thomas Hübl (Hübl, T. (2020). Healing Collective Trauma: a process for integrating our intergenerational and cultural wounds. Boulder: Sounds True.), which is a method to restore fragmentation by addressing and integrating individual, ancestral and collective trauma. This paper focuses on one methodological consideration for building a new culture through the integration of collective and intergenerational trauma, which is a framework based on collective trauma research in psychology, sociology, and quantum social science.

## Introduction

Collective and intergenerational trauma is not only psychological phenomenon discussed in psychology and psychotherapy, but also social phenomenon researched in sociology and other social science. This phenomenon should be described from the perspectives of both methodological individualisms and collectivisms, and both psychological and social pathology. For this, we need more holistic interdisciplinary approaches which can disentangle the snarled collective and individual entity. One approach, which was originally developed for international relations in political science, is ‘quantum social science’. In this paper I use the quantum social science approach to describe the individual psychological and collective sociological pathology of collective and intergenerational trauma, and explore the functions and mechanisms of this phenomenon.

This approach conforms to the theoretical assumptions of quantum social science of Alexander Wendt and collective trauma theory of Jeffrey Alexander and Thomas Hübl, who formulate their transformative scientific perspective as a means of coordinating our behavior to contribute to a more robust process of culture-building. This approach is organized in three sections: quantum social science approach (Sect. 2), its application for collective and intergenerational trauma (Sect. 3), and practical instruments for collective trauma integration (Sect. 4).

## Quantum Social Science Approach

According to Feynman (1964), quantum mechanics is the fundamental theory of quantum physics which provides a description of the physical properties of nature at the scale of atoms and subatomic particles. The most important concept is ‘quantum coherence’ which proposes that all objects have wave-like properties. If an object's wave-like nature is split in two, then the two waves may coherently interfere with each other in such a way as to form a single state that is a ‘superposition’ of the two states. ‘Superposition’ is the ability of a quantum system to be in multiple states – wave and particle – at the same time until it is measured or observed. How particles behave either as waves or as particles depends on whether they are being observed. The transition process of a quantum system from a superposition of states to a component state is also known as collapse of the wave function or ‘quantum collapse’. How observation causes this collapsing, and what constitutes observation (measurement) are currently unresolved questions in quantum mechanics. There are many interpretations of quantum mechanics. One of these is a hypothesis that consciousness collapses the wave function, and this consciousness-hypothesis has been applied in social science. For example, as Zohar and Marshall (1994) propose, the creative combination of individual identities preserved and enhanced within a collective identity is made possible through the wave/particle duality of quantum reality. They write:

The particle aspect is the individual aspect. Its characteristics are determinate. We can locate particles in space and time, we can focus on them and measure them. They are separate and relate mechanically. The wave aspect is indeterminate. It is a spread of possibilities located everywhere in space and time. We cannot pin waves down; they have no boundaries. Each wave overlaps and combines with all other waves (p. 109).

Recently many physics and social science researchers who consider how concepts, methods, and understandings from quantum physics relate to societal issues are establishing ‘quantum social theory’. One such approach is Alexander Wendt´s quantum conscious hypothesis, which is briefly summarized below.

Wendt (2016) illustrates how and why a quantum social science that emphases non-locality, indeterminism, non-dualism, and holism challenges metaphysical assumptions of the classical worldview (locality, determinism, dualism, and atomism). He argues that quantum mechanics are not irrelevant to the macro scale. He also explains social life from a quantum perspective and insists that “human beings and therefore social life exhibit quantum coherence – in effect, that we are walking wave functions” (p. 3). On the one hand, his argument is speculative, based on a thought experiment that presupposes quantum coherence in the brain, but on the other side, it is a self-evident that “human beings have material bodies that think and interact with each other through thought, voice, sound, sight and touch, all of which seem indisputably subject to the law of physics” (p. 11).

The core proposition for quantum social theory by Wendt can be summarized as follows: Due to ‘direct perception’ or ‘pure experience’ (measurement), an agent can be aware of affordance which is an action possibility formed by the relationship between an agent and its environment. This process of becoming aware of affordance – ‘intra-action’ between subject (agent of observation) and object (object of observation) – can bring out an agent´s Will, which is expressed in a speech act with perlocutionary force (quantum collapse). Wendt adopts quantum physics to establish a new social theory which is different from a classical worldview such as materialistic, deterministic, mechanistic and individualistic.

As mentioned above, there is a long tradition of social theory, including Whitehead, who “was motivated by the quantum revolution to articulate a post-classical cosmology to display Newtonian cosmology” (Allan, 2018, p. 98). Whitehead and others from this tradition such as Nietzsche, Dewey, Bergson, Tarde, Deleuze, and Nishida proposed metaphysical views of society and the universe that were inspired by the natural sciences. Wendt, who also belongs to this tradition, strives to create not a purely metaphysical, but a logical, post-classical account of human action by drawing from quantum mechanics.

Wendt´s methodological approach is to draw out the epistemological lessons of quantum theory for social science by essentially trying to guess what the relevant complementary variables would be. In writing this paper, I am interested not only in analogies but also in the widely applicable psychological and sociological issues such as personal healing and societal transformation, cultural process, the nature and function of bodies, the nature of identities, and consciousness and the unconsciousness.

## Collective and Intergenerational Trauma

There are some widely acknowledged researchers and authors who have contributed definitions of collective trauma, e.g. Volkan (1997), Neal (1998), Alexander (2012), Aydin (2017), Hirschberger (2018), Hübl (2020). They all belong to the “enlightenment thinkers” (Alexander, 2012) who suggest “trauma is a kind of rational response to abrupt change, whether at the individual or social level” (p.2). Among these scholars, there are various adjectives modifying this trauma category, which the literature includes in related terms such as: collective trauma, social trauma, cultural trauma, historical trauma, intergenerational, and transgenerational trauma.

For Alexander (2012), “cultural trauma occurs when members of a collectivity feel they have been subjected to a horrendous event that leaves indelible marks upon their group consciousness, marking their memories forever and changing their future identity in fundamental and irrevocable ways” (p.1). He reports on his empirical research and interpretation concerning the Holocaust, Hiroshima, Nanjing, and the India/Pakistan conflict, showing that “this new scientific concept also illuminates an emerging domain of social responsibility and political action” (p.1). By constructing cultural traumata, social groups and national societies identify not only the existence and source of human suffering cognitively, but also take on some significant responsibility for it. Hirschberger (2018) suggests that “the tragedy is represented in the collective memory of the group, and like all forms of memory it comprises not only a reproduction of the events, but also an ongoing reconstruction of the trauma in an attempt to make sense of it” (p.1). Collective memory of trauma is not the same as individual memory or trauma because “collective memory remains beyond the lives of the direct survivors of the events, and is remembered and narrated by group members that may be far removed from the traumatic events in time and space” (p.1).

Hübl (2020) uses “intergenerational trauma” which “refers to the effects of serious, untreated trauma which has been experienced by one or more members of a family, group, or community, and has been passed down from one generation to the next through epigenetic factors” (p.66). Hübl (2020) defines “historical trauma” as a wider, diffused intergenerational trauma:

When we think of historical trauma, we think about the painful and long-lasting consequences of war, imperialism, colonization, domination, subjugation, occupation, enslavement, interventionism, and hegemony. It is a force that often proliferates as a result of cultural, political, racial, ethnic, religious, gender and/or sexual extermination, suppression, or systemic intolerance. (p.67)

Cultural, collective, intergenerational, or historical trauma have caused “a large group to face drastic losses, feel helpless and victimized by another group, and share a humiliating injury” (Volkan, 1997, p.8). A large group does not choose to be victimized or suffer humiliation, but some members of the group may choose unconsciously to include a trauma narrative of the event as part of their identity. Volkan (1997) discovered that “while groups may have experienced any number of traumas in their history, only certain ones remain alive over centuries”, which is called “chosen trauma”. Chosen trauma “is linked to the past generation’s inability to mourn losses after experiencing a shared traumatic event, and indicates the group’s failure to reverse narcissistic injury and humiliation inflicted by another large group, usually a neighbor” (Volkan, 1997, p.7). The chosen trauma is “woven into the canvas of the ethnic or large group tent, and becomes an inseparable part of the group’s identity” (p.10). The chosen trauma persists as dangerous memory which is “disruptive to taken-for-granted assumptions about a group´s identity” and “threatening to official memory and the vested interests of the nation state as well as its investment in essentialist identities” (Bekerman & Zembylas, 2012, p.196). Subsequent generations of trauma survivors who never witnessed the actual events may remember the events differently than the direct survivors, and then the construction of these past events through chosen trauma may take on a different shape and form as the narrative is passed down.

### Entanglement of Ancestors´ Chosen Trauma and Descendants´ Identity

Victims (and their ancestors) of mass atrocities such as genocide and war choose, memorize, and narrate these atrocities as trauma, influencing their descendants emotionally, mentally, or somatically. Chosen trauma of historical events is transmitted intergenerationally (intergenerational trauma) and affect subsequent generations (transgenerational trauma). Chosen trauma that ancestors experienced has demonstrated social and psychological impacts on the current generation (e.g., Pennebaker et al., 1997). How this transmission works is explained in psychology, social constructivism, and social epigenetics.

Historical traumatic events destroy “the basic tissues of social life that damages the bonds attaching people together and impairs the prevailing sense of communality” (Erikson, 1976, p.153). Sometimes the community does not exist as an effective source of support any longer, so that “‘We’ no longer exist as a connected pair or as linked cells in a larger communal body” (Erikson, 1976, p.154).

The world-altering impact of World War II is one such example. In 1945, two atomic bombs killed 246,000 people in Hiroshima and Nagasaki. Survivors experienced extreme physical and emotional distress (Lifton, 2012; Oughterson & Warren, 1956). A longitudinal study (Amano et al., 2021) reports that over the next 60 years (1950–2009), 1150 suicide deaths were recorded among 120,231 participants (23.6 per 100,000 person-years). Among those < 25 years of age at the time of the bombings, he noted an increased suicide risk. This study supports many narratives of Japanese survivors who witnessed that several young people in Hiroshima and Nagasaki committed suicide by jumping in front of a train immediately after the bombing after losing their families, friends, and communities.

Vignoles et al. (2006) suggest that collective trauma may facilitate the construction of various elements of meaning and social identity: purpose, values, efficacy, and collective worth. These effects of trauma on the construction of collective meaning may increase as time passes following the traumatic event (Klar et al., 2013). This occurs, as Hirschberger (2018) argues, “because the focus of memory shifts from the painful loss of lives to the long-term lessons groups derive from the trauma” (p.3). This argument can be understood as absence of reflexivity to respond collective trauma. One has paid little attention to the experienced pains and deaths of the victimized ancestors of her group or of those who suffered under her perpetrator ancestors, because her group, community, or state has made enormous efforts to rebuild a culture. This rebuilding process of a culture socialized its members into forming a coherent social identity by constructing *meaning*.

Social epigenetics studies the way experiences and situations are biologically incorporated, examining how they may leave a biological marker on the body via epigenetic mechanisms (Dubois & Guaspare, 2020). The argument that the genome appears to be modified or altered is made by some researchers who report: Intergenerational trauma is not only passed on through sociocultural environments, but also through DNA (Pang, 2015); new findings suggest that trauma experienced by survivors during the Holocaust may be passed down in genes (Khurshid, 2015; Yehuda et al., 2008); A number of research finds that those who have been traumatized around the time of conception can pass on a DNA code to their offspring that results in a higher vulnerability to stress in their molecules, neurons, cells, and genes (Giang, 2015); Research has revealed that when people experience trauma, it changes their genes (Blades, 2016), etc. However, none of this research claims to have proven positively that the genome can be modified. Social epigenetics is a developing research discipline that “makes it possible to attest that this past, far from being finished, is still alive and well in a degraded way of life” and can “reaffirm a collective identity based on sharing the same biological and social condition, both in the past and present” (Dubois & Guaspare, 2020, p.168). In this way, social epigenetics can become a societal resource for finding and defining the collective identity of multiple generations who share the same collective history of adversity.

Many trauma therapists, researchers, and thought leaders insist that individual trauma must be viewed through the context of social trauma. For Hübl (2020) social and individual traumas are entangled and conformed as “a kind of collective trauma bonding” (p.91). Entanglement or bonding, often unconscious, influences collective and individual behaviors in a post-traumatized society. The unaware entanglement can be interpreted as ‘a social form of Post-Traumatic Stress Disorder (PSTD)’ (Lerner, 2012), which exhibits social symptoms like:

- *Social pressure for silence*: a traumatized society has become collectively withdrawn from public emotional expression and fatalistic in outlook. “Silence about the trauma is enforced by social pressure because it would be too painful to re-experience the original terror or shame” (Rinker & Lawier, 2018, p.152).
- *Pseudo-safety*: “The dominant community in such a traumatized society, having failed to work through their own past trauma, empowers itself by over-subjugating the oppressed. The dominant community tries to achieve a sense of pseudo-safety by force and justifies inhumane treatment of the oppressed by dehumanizing them socially and economically” (Rinker & Lawier, 2018, p.152).
- *Conflict-in-process*: In post-traumatic communities, people live the social legacies of long-term historical trauma, these communities are still dealing with the traumatic historical events in the context of ongoing violence (i.g., Sandole, 1998).
- *Vicarious trauma*: certain descendants of traumatized society have not been directly exposed to violence, but exhibit the symptoms of having experienced the trauma. Furthermore, this trauma is constantly re-triggered and reinforced by virtue of proximity to a perceived ‘enemy other’ (Rothbart & Korostelina, 2011, p.28), or what Volkan (1988) called ‘suitable targets for externalization’.

Those who experience these kinds of a social form of PTSD reenact not only an unconscious inter- and transgenerational transmission of a chosen trauma, but also transform it into guiding narrative of their identity.

This narrative identity as descendants of victims draws disparate tribes together over years, ultimately creating a psychological framing around transgenerational and intergenerational victimhood. For example, the tragic event in Hiroshima/Nagasaki has been experienced as collective trauma by survivors in both cities, and transmitted inter- and transgenerationally through the post-war nationalist political narrative, which states, Japanese are not perpetrators, but victims. This narrative identity has been shared unquestionably by almost all Japanese since 1945. (cf. Section 4). The collective and individual symptoms which result from the unconscious influence of trauma are clearly evident in post-traumatized societies. The invisible entanglement between chosen trauma and narrative identity acts to reproduce post-traumatized, socio-cultural dysfunctions such as human rights abuses, lack of political transparency, economic instability, polarization, and political extremism, among other issues.

### Entanglement of Ancestors´ Perpetration and Descendants´ Historical Unconsciousness

The effects of collective trauma on the construction of meaning is not limited to the victim group that needs to reinvent itself and reconstruct all that was lost, but also impacts the perpetrator group which must redefine itself and construct a positive moral image in light of the atrocities it committed (Hirschberger et al., 2016; Imhoff et al., 2017; Shnabel & Nadler, 2008). Hirschberger (2018) sheds more light on how collective trauma is experienced by the perpetrators. Reminding them of the responsibility of their group for past misdeeds leads to derogation (Castano & Giner-Sorolla, 2006) and to negative attitudes toward the victim group; it leads to a defensive attempt to protect the group by minimizing the historical crime (Doosje & Branscombe, 2003), distorting the memory of the event (Dresler-Hawke, 2005; Frijda, 1997; Sahdra & Ross, 2007), and justifying in-group behavior (Staub, 2006). Members of perpetrator groups often display blind spots in their memory of the event in order to eliminate inner conflict (Dalton & Huang, 2014; Frijda, 1997), or deny the ongoing relevance of the past by demanding historical closure on this chapter in history (Hanke et al., 2013; Imhoff et al., 2017).

Orange (2017) discusses critically about perpetrators´ ‘blind spots’ in the context of climate change which causes climate injustice. “Our profound individualistic egoism prevents us from noticing both what we are doing to each other and our planet as well as to the ways we are beneficiaries of the slave system, of colonialism, and of carbon-dependent industrialism” (p.13). This unconsciousness, which might be a part of our ‘blind spots’ as historical trauma, blinds us further to egoism´s devastating consequences. Orange (2017) calls perpetrators´ blind spots “historical unconsciousness”. In the U.S. the historical “unconsciousness is silent about the U.S. history of settler colonialism, ignorant and mute about our crimes of chattel slavery and racial domination, neither governments nor citizens can seriously tackle climate injustice until we confront this 400-year history” (p.37). Not only climate injustice but also many other social problems in the world can be rooted in a shared historical unconsciousness. Orange (2017)´s critical voice is very sharp and loud: “Blindness to our ancestors´ crimes, and to the ways we ‘whites’ continues to live from these crimes, keeps the suffering of those already exposed to the devastation of climate crisis impossible for us to see or feel.” (p.39).

Particularly since 2015, Europe has seen an increase in refugees arriving from former European colonies like Syria, Afghanistan, and various African countries, fleeing from poverty, war, and terrorism perpetrated by groups such as ISIS in Syria, Al Qaida, and the Taliban in Afghanistan, and Boko Haram in Nigeria. Moreover, considerable scientific evidence supports a correlation between this surge in numbers and the escalating environmental degradation, consequences of climate change, and international tensions surrounding these regions. For example, Burke et al. (2015) found evidence for a correlation between higher temperatures and the occurrence of armed conflict in sub-Saharan African countries, suggesting climate change will lead to further escalation. Hsiang et al., (2013) examined 60 studies and found that climate change was linked to conflict over a broad span of time and geography. Kelley et al. (2015) concluded that human influence on the climate system are implicated in the current Syrian conflict. Nevertheless, no study has yet described a mechanism by which climate change leads to conflict. Climate change, particularly global warming, is for the most part caused by our industrialization and modern consumer behavior, leading to dangerously high CO^2^ emissions. For example, there are ongoing international research projects that seek to explore public understanding and behavior in relation to meat and dairy consumption and its impact on greenhouse gas emissions. Too much meat and dairy consumption does not only have a serious impact on climate, but also on poverty, conflict and, consequently, displacement. Unhealthy eating habits and lifestyle trends in western societies are leading to widespread physical and mental illness, both locally and globally, not only concerning one’s own circle of friends and family, but causing a ripple effect across the globe (Grimm, 2020; Hicks, 2011). The most pressing global crises, which include unprecedented population displacement, wars and genocides, poverty and hunger, and environmental degradation, encompass numerous socio-economic factors and dynamics. However, many of these crises seem to be interrelated, and connected to climate change in particular, which, in turn, is inextricably linked with and caused by mundane, everyday behaviors (Welzer, 2017). Victims of these crisis are descendants of people oppressed by European colonialists, and perpetrators of these crisis are descendants of former European colonialists. This victim-perpetrator-constellation remains without role changing as Orange (2017) refers:

Together with the colonialist past we all share, this history of slavery and its ongoing effects, of which we rarely speak, blinds us to the mystery that our carbon-and-methane-spewing lifestyles are creating in the Global South. We are repeating. (p.45)

So far as perpetration of our ancestors has not been atoned sincerely by them and their descendants, the descendants are possessed by historical unconsciousness and their behaviors have negative impacts on the descendants of historical victims consequently. In this way ancestor who were perpetrators become entangled with the historical unconsciousness of their descendants, and perpetration and victimization continue to repeat non-locally and timelessly.

### Superposition: Culture and “Dark Lake”

Culture influences its integral parts and institutions in which individuals are enculturated, acculturated, and interact with each other. In this socialization process, their cultural identity is constructed and protected by the transgenerational transmission of a mental representation of a traumatic historical event (chosen trauma) (Volkan, 2001). This transmission process provides values, goals, and ideals to give individuals´ lives a desired meaning and direction and to enable them to form a coherent, healthy, and strong individual and cultural identity. The intelligence of this collective trauma transmission is to banish the trauma by integrating it into the identity of a culture in such a way that it ceases to paralyze that group or community.

Aydin (2017) suggests that “if the collective trauma is not dealt with, it will ultimately completely define the identity of a culture. Therefore, a culture will entirely coincide with its history, or even worse: with one dark page of its history” (p.131). The unintegrated collective trauma “disorganizes the community´s psychic economy because the beliefs, values, expectations, and ideals that are part of its cultural identity are radically shaken, challenged, and disrupted” (p.129). This inability to reconcile and resolve a dark episode of history can “disrupt the organization of the values, expectations, and ideals of a victimized group to such a degree that it no longer can provide sufficient orientation and self-esteem” (Volkan, 2001, p. 87). Consequently “the further development and flourishing of a group’s cultural identity can be severely disturbed and even completely obstructed” (Aydin, 2017, p.129).

If the unintegrated collective trauma is too big to digest, it damages consequently the resilience of a culture, which hinders further growth and flourishing. When the trauma cannot be integrated, it remains invisible and becomes an invisible shadow as part of the cultural identity of the victims and their descendants. According to Aydin (2017), “they are unable to integrate the trauma into their cultural identity and, at the same time, they can never let go of what has happened”.

It becomes a monster that haunts them unceasingly, a monstrum, something beyond normal human bounds that cannot be categorized and identified and persistently disrupts their mental order. It is inconceivable but, nevertheless, ever present. (p.131)

This ‘monstrum’ is hidden in a dark lake which we can perceive as a shadow of culture, but cannot grasp and recognize by language. The language we’ve acquired in our socialization process is not adequate to describe it. Hübl (2020) explains:

In the trauma field, large portions of energy are strongly dissociated and suppressed in shadow. Their energy resides in the dark lake, largely invisible to members of the society, even while the symptoms they produce are widely manifested throughout. The nervous system of the next generation is born into and raised within that field, which is invisible; its effects and structures become wired and are taken as “normal.” This traumatic-state conditioning makes it difficult for any person living within that community to realize a felt sense of its dark lake, even after such a native comes to cognitively recognize its existence. (p.92)

The ‘dark lake’ in which the ‘monstrum’ is hidden reappears with fears, expectations, fantasies and when “a chosen trauma is reactivated” and “time collapses occurs” (Volkan, 2001, p.10). ‘Time collapse’ is expressed psychologically as ‘flashbacks’ in which an individual has a sudden, usually powerful, re-experiencing of a past experience or elements of a past experience. ‘Time collapse’, on one side, makes both conscious and unconscious connections between the past trauma and a contemporary threat and “magnifies the image of current enemies and current conflicts”, so “the sense of revenge becomes exaggerated” (Volkan, 2001, p. 10). On the other hand, ‘time collapse’ presents an opportunity to discover monstrum´s thoughts, feelings, hidden truths, and incomplete movements which still remain fixated in space and time. Beginning with this recognition, individuals, families and entire cultures can then find a path to completion.

Without time collapse, the ‘dark lake’ remains invisible and unrecognizable. This ‘dark lake’ extends beyond our notions of space and time, occupying more than one position at any moment of time, like ocean waves that move along a stretch of beach. This wave movement overlaps with others and can occupy the same position at any moment of time. During the period of when the invisible ‘dark lake’ ensues, before the sense of time collapses, only the context in which the monstrum is hidden is recognizable – it is culture. Culture is localized objects that occupy a given location at each moment in time. The phenomena of the ‘dark lake’^1^ and culture are distinct, but hold mutually exclusive characteristics. The phenomena is similar to a ‘superposition’ which is the ability of a quantum system to be in multiple states – wave and particle (see Sect. 4.1.).

**Fig. 1:**
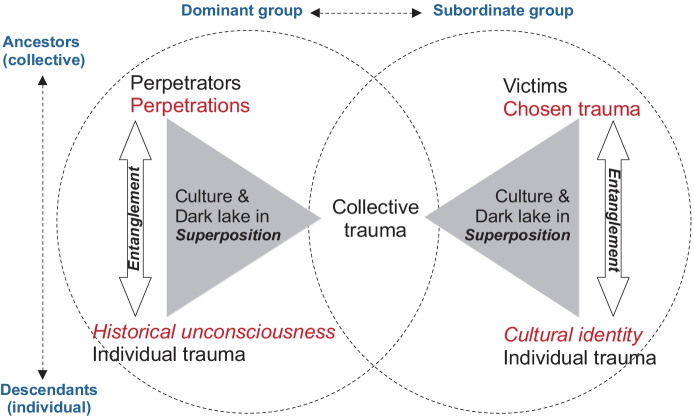
Entanglements and superposition in collective and intergenerational trauma

Entanglement and superposition are related and positioned in the matrix of victims, perpetrators, and ancestors (Fig. 1).

A group of people who were victimized by another group, for example in a genocide or a massacre, focuses on certain parts of this terrifying event which becomes their ‘chosen trauma’. The event has been transmitted as ‘chosen trauma’ intergenerationally from ancestors to descendants in this group. This transmission process occurs through identity politics which are manifested via political and societal power. The group operates in this manner to establish a stable foundation for their state and community. Descendants of victims are socialized under the influence of these identity politics and are identified with a specific cultural identity of the group. Individual cultural identity of each descendent becomes entangled with the chosen trauma of their ancestors (collective).

However, on the other side the perpetrators of these victims experience a different process in the transmission of their perpetrations. The quality of these experiences can be interpreted as glorifying or they may forget their crimes. This process is typically enacted systematically in the form of identity politics inter-generationally from ancestors to descendants. The results are that descendants become unconscious of the perpetration committed by their ancestors. In this way, the historical unconsciousness (individual) of descendants is entangled with the perpetration of their ancestors (collective).

These two types of entanglements occur within the respective culture of each group. Culture is dynamic, and changes in its cultural process by providing an important context for entanglements. While culture is seen as “a consequence and a function of the social dimension of human life,” cultural process is “a consequence and an expression of human consciousness of the world and intentionality toward the world” (Lankshear, 1997, p.13). In cultural process “individuals are identified or identifiable as members of socially meaningful groups or networks and as players of meaningful social roles” (Gee, 1990, p.142–3). Cultural process is mediated by using language as a ‘medium’ and a ‘broker’ (Lankshear, 1997, p.21)^2^ by which human individual and group identities are constructed. With language as medium, our behaviors and relationships to other human beings and the world become conscious and intentional; they become objectified as an object of interaction. As a ‘broker’ is an agent acting as a mediator between dominant and subordinate groups by enlisting the services of language, language as a ‘broker’ can disentangle the invisible knots between ancestors (collective) and descendants (individual). If the cultural process of disentanglement through language as a ‘broker’ enables the superposition collapse, the ‘dark lake’ becomes visible and sensible.

## Collective and intergenerational trauma integration: A quantum approach

Collective and intergenerational trauma hinders healthy societal development and damages individual mental health. Individual trauma should be treated adequately by competent psychotherapists and groups facilitated by those who are trained in these processes. Optimal results will be achieved if experts in therapy collaborate with social scientists such as sociologists, historians, and political scientists who are capable of analyzing the phenomena of collective trauma. Without their skills and insights, it is not possible to pathologize social phenomena like collective and intergenerational trauma. Hiroshima was, for example, a mass atrocity that many Japanese experienced on August 6, 1945, and has been a “collective memory” for 75 years (Bekerman & Zembylas, 2012). This memory was repeatedly told and represented in society and culture. This process of storytelling and meaning making is “a complex and multivalent symbolic process that is contingent, contested, and sometimes highly polarizing” (Alexander, 2012, p. 8). This process, which is sometimes manipulated politically, influences our narrative and cultural identity building (cf. Aydin, 2017).

Sociological framework for process analysis provides four critical dimensions of representations as an analytical tool: (1) “the nature of the pain”; (2) “the nature of victim”, (3) “relation of the trauma victim to the wider audience”; (4) “attribution of responsibility” (Alexander, 2012, p. 8). With this information in the context of ‘social pathology’ (Kettner & Jacobs, 2016), a ‘social diagnosis’ (Richmond, 1917) can be made and a proper ‘social therapy’ (de Maré, 1991) can be selected. A social therapy which is selected wisely based on the social pathology and to diagnose the suffering or the experiences of a community gives their trauma a place in their cultural identity. The therapy also actively integrates the trauma by reinterpreting the originating event. The process is also integral to identifying and framing the monstrum in the ‘dark lake’. This process of reinterpretation is “an essential requirement for successful sublimation**”** (Aydin, 2017, p.135). It means the transformation of negative and destructive emotions and energy caused by a traumatic event into positive, constructive emotions, actions, and behaviors.

According to Thomas Hübl, one of the most effective approaches to collective trauma integration is the cultivation of coherence and resilience in groups, followed by a process of consciously turning towards and witnessing the emerging sharing of individual, ancestral, and collective trauma experiences. As people gather around a shared intention, they have more resources available and can integrate the pain, bit by bit, that could not be processed before. In this process they can sense the ‘dark lake’ as Hübl (2020):

When we gather for the purpose of integrating collective trauma, we might not see this dark lake with our eyes, but with sensitivity and attunement, we can sense its distinctness and feel its forms. The collective shadow of Argentina, the United States, Israel, or Germany each feels particular to the place and to the people who live and have lived there. (p.148)

The ensuing release leads to mindset shifts and behavioral change—an increase in compassionate and collaborative ability, creativity and innovation and a decrease in isolation, polarization, and separation. Based on his experiences as a facilitator who has worked with thousands of people, Hübl developed the “Collective Trauma Integration Process (CTIP)” whose goal is to release and thereby integrate the trauma that is stored both within individuals and within groups of people who share a similar trauma pattern in their collective unconscious. According to Hübl (2021, p.11) the collective trauma integration process (CTIP) model has six core stages:

1. Synchronizing and resourcing
2. Meeting the landscape
3. Becoming a conduit
4. Listening to the field
5. Integrating and restoring
6. Transforming and meta-learning

These six stages in the CTIP which takes place in a group of ca. 20–150 people guided by one or two facilitators might enable participants to break entanglements and the superposition to collapse through ‘redemptive measurement’ and ‘expressive measurement’ (Fierke & Mackay, 2020). In the following sections these two ‘measurements’ will be explained more detail.

### Measuring Trauma

Quantum social science by Wendt (2016) hypothesizes that language (speech act) causes breaking of entanglement and collapsing of superposition at the macroscopic level, which is society. The act of speaking using language is a form of measurement that impacts what is observed and brings about a collapse from potential meaning into an actual one. Fierke and Mackay (2020, p.452) note that “the collapse starts with communicative intent (the decision to communicate one meaning rather than another), which depends also on the listener, whose understanding will depend on how what is said interacts with a memory of words and their association”. Besides language as ‘expressive measurement’, Fierke and Mackay (2020) explore the quantum notion that “to ‘see’ an entanglement is to break it” (p. 450) and to witness unacknowledged past trauma is proposed as ‘redemptive measurement’. These two types of measurement are characterized with a common basic function – discursive practices, as communicative practices of engagement with, and as part of, the world in which we live. According to Barad (2007) discursive practices are “specific material reconfigurings through which objects and subjects are produced” (p.148). Discursive practices are specific agential intra-actions that “produce determinate boundaries and properties of “entities” within phenomena” (p.148). ‘Expressive measurement’ and ‘redemptive measurement’ enable us to detangle the entanglement between ancestors (collective) and descendants (individual) and to determinate a boundary between two dimensions (culture and ‘dark lake’) within the world of our being, becoming a collapse of superposition.

### Witnessing as Redemptive Measurement

Fierke and Mackay (2020) explain that a redemptive measurement is to see that “which is hidden or unseen and to give it a place of belonging within the relational field […] so that the traumatic entanglement is broken and a more positive relationality can begin to be restored” (p.459). To see or to witness as redemptive measurement transforms a collective trauma field into a corrective trauma narrative, in which “the suffering is seen and the trauma loses some of its power” (p.459).

‘Global social witnessing’ (GSW) was originally proposed by Hübl and Ury (2017) and was developed as a practice of redemptive measurement. Matoba (2021a) defines this method as “the emergent human capacity to mindfully attend to global events with an embodied awareness, thereby creating an inner world space mirroring these events” (p. 59). Matoba (2021b) further defines the core concept of witnessing as a process to perceive unconscious interconnectedness as entangled individuals and to sense a non-local correlation between subject and object. This process “results in felt-oneness both for subject and for object” (p.14). In a GSW process, participants are invited to connect with a historical traumatic event by first learning about the context and the facts of the event and then engaging with it through images, such as studying an image of the event. The intention behind this process is to allow oneself to be affected by the event, to become aware of phenomenal impressions on various levels (mental, emotional, somatic, relational, etc.), and to attentively stay with these impressions and their unfolding within one's awareness. This process can be facilitated with the following prompts: (1) Witness what happens in your mind; (2) Witness what happens in your emotion; (3) Witness what happens in your body; (4) Imagine you are in a dialogue with a person affected. What would this person ask you? And how would you answer their questions? (5) Witness what happens in your mind, emotion, and body in response to this imagined dialogue. Matoba (2020a) improves ‘global social witnessing’ conceptually as an educational tool for awareness-based systems change by drawing on Emmanuel Levinas’s philosophy of relational responsibility, and focusing on transformative, systemic learning.

### Transparent Communication as Expressive Measurement

What is witnessed is expressed in language. Fierke and Mackay (2020) call this language usage ‘expressive measurement’ because “language expresses a form of ‘seeing’ by the observer as wave functions collapse” (p.453).

Some researchers and trainers of collective trauma integration use a systemic constellation, which was developed by Bert Hellinger to disclose the hidden patterns unknowingly influencing thoughts, behaviors, and emotions in a system such as a family. Fierke and Mackay (2020) report that in their systemic constellation experiment the participants who occupy positions in a relational system can represent the bodily sensations, feelings and impulses of someone who is experiencing collective traumatic memory. Their collective trauma becomes visible as phenomenon through this ‘expressive measurement’ which manifests as a past collective experience of suffering through representatives within the experiment, who experience the affective resonance surrounding this past.

These articulations express a form of wave-function collapse and a pattern of diffracted entanglement, by which the attributes of the system become visible or ‘seen’ as the vibrational frequencies surrounding a particular space, and the affect that arises from it, are transformed into language and thus became available for analysis. If language use is a measure of wave-function collapse, the language arising from a relational system becomes a measure of a non-linear historical trauma field. (p.458)

The communicative quality of expressive measurement should have illocutionary and perlocutionary force, with which wave-function superposition collapses (see Sect. 2, cf. Matoba, 2021b). Wendt (2016) argues that “the ‘force’ that collapses the wave function is Will” which “is the locus of mental causation, the ability of the mind to direct the behavior of the body” (p. 263).

One intra-active communicative method for expressive measurement is ‘transparent communication’ developed by Thomas Hübl. According to Hübl (2020, p.220) “humans are capable of entering into more aligned sphere, a collective state of being and awareness”. Transparent communication is to communicate from the inner sphere and to speak from the energy and information of anther. This communication enables us to access a more extensive level of information in our lives and to move beyond the interpretation (understanding) of humans as objects in the physical world and thus experience humans from within. This method helps us to acknowledge the true cause of many conflicts, looking beyond the symptoms to the root of the problem.

In CTIP, what is witnessed as a practice for a redemptive measurement is expressed by practicing transparent communication as expressive measurement. For example, one participant senses bodily tightness and feels a difficulty in breathing when she listens to a discussion about a terrible event that occurred in her country. She can witness the event and can express verbally how she witnesses it cognitively, emotionally, and somatically. This measurement process should be facilitated by competent facilitators who guide participants to implementing intra-action. The facilitator guides the group in observing thoughts, cognition, emotions, feelings, and body sensations as integral parts of a phenomenon shared with the other group members.

### Group Coherence for Intra-Action

In the collective trauma integration process (CTIP), two types of measurement – redemptive and expressive measurement – are enacted by participants in a group. Just as physical measurements such as length, weight, and speed require an apparatus, redemptive and expressive measurements are conducted by apparatuses too. In the context of CTIP, each participant can be understood as an apparatus of measurement that depends on other apparatuses in the group. This interdependence of apparatuses has been discussed as ‘agency of observation’ among many quantum physicists and philosophers, starting with Nils Bohr and Werner Heisenberg. In social contexts such as CTIP, and in framing this as a quantum discussion on the nature of apparatus, an apparatus of redemptive and expressive measurement is not limited to individual cognition, emotion, and sensation. The apparatus should be regarded as holistic collective performative action, a group of participants as an apparatus. For Barad (2007), “apparatuses are not static laboratory setups but a dynamic set of open-ended practices, iteratively refined and reconfigured” (p.167). Apparatuses enact ‘agential cuts’ that produce determinate boundaries and properties of entities within collective trauma phenomena. These phenomena are exhibited in a superposition ‘culture/dark lake’ which are intra-acting as ontologically inseparable components. Barad (2007) writes “agential cuts are at once ontic and semantic. It is only through specific agential intra-action that the boundaries and properties of “components” of phenomena become determinate and that particular articulations become meaningful” (p.148). This “specific agential intra-action” – redemptive and expressive measurement – is only possible through an apparatus with high quality, which Hübl (2021) calls ‘group coherence’.

Through individual, relational and we-space awareness, we can build a certain level of group presence or coherence. Through certain relational practices and competencies, but also through our ongoing integration work, we can continue to upgrade a group’s capacity to develop higher group coherence. The group participants weave together a certain atmosphere or group field, that is an expression of the process awareness that this specific group is able to hold. This becomes a resource that enables us to meet the hidden fragmentation within the system. Higher group coherence amplifies our innovation and/or integration capacity. Unintegrated fragments in a group are unconscious zone that are waiting to become integrated structures of consciousness and that provide learning. The frozen past is integrated into presence and becomes future potential. (p.2)

The term ‘hidden fragmentation’ refers to the ‘dark lake.’ which is one existing potentiality of a superposition culture/dark lake. As the superposition culture/dark lake is visualized in Fig. 1 above, culture is present in entanglement of the collective/ancestors and individuals/descendants on both sides of victims and perpetrators. Culture is a crossroad of collective-individual line and victims-perpetrators line. If the crossroad of these two lines can be lightened by detangling the entanglement of collective/ancestors and individuals/descendants on the both victims´ and perpetrators´ sides through intra-active measurements, the shadow of the crossroad – ‘dark lake’ – can be made visible. This process leads to a collapse of superposition in the integration of collective and intergenerational trauma.

## Concluding Thoughts

In a culture constructed by invisible entanglements (when there is no conscious untangling) between collective/ancestors and individuals/descendants, we cannot have any reflexivity to respond to collective trauma, but we are also unaware of this absence of reflexivity, which is the nature of unconsciousness. The concept of reflexivity itself cannot work within a complex society that experiences global crises (e.g. waves of refugees, wars, and genocides, poverty and hunger, environmental degradation). The complexity of these crises involve numerous variables and impacts, but they all seem to be interrelated. They are especially related to the ubiquitous nature of climate change which is clearly embedded in and derived from our behavior in daily life (Welzer, 2017). In this context of our self-traumatization, while being unaware of the absence of reflexivity, we (subject) try to transform the world and the earth (object) through interactions to actualize the world as we wish to perceive it. The self-traumatization makes us blind, so that we can know but not sense the fact that subject and object are rooted in causality and do not exist as separate, independent entities. When there is a distinction between subject and object, knower and known, the knowledge gained through reflexivity doesn’t allow a perception of the whole, but only half of reality.

Without perceiving the other half of reality we cannot discover a comprehensive, wholistic solution to complex problems, nor can we fully integrate our collective traumas. To see another half of reality we need special measurements and apparatuses which enable ‘diffraction’, which is defined in physics as the blending of waves (i.g. electron beam and neutron beam) around the corners through a slit into the region of geometrical shadow of the slit. Since Barad (2007) applied this physics terminology to the social sciences, diffraction has emerged as a concept and methodology through opposing reflexivity. While reflexivity refers the reflection of objects held at distance, with a preexisting determinate boundary between subject and object, diffraction is defined by Barad (2007, p.89) as “making differences from within and as part of an entangled state” whereby “subject and object do not preexist as such, but emerge through intra-actions”. Collective and intergenerational traumata which are phenomena materialized by entanglement and superposition can be integrated in a performative process by diffraction and intra-actions.

In the CTIP, vocabulary from quantum social science can be used to enhance the experience of participants in their understanding of the process. Most importantly, the participants are enacting the language of quantum social science by embodying their diffractive and intra-active meanings. As they do this, the individuals (subject) and their collective traumata (object) can be enacted in a new way, as part of a new vision for culture. In this way, CTIP is one example of a quantum social science approach to collective trauma, highlighting the ways in which trauma can be rationalized, managed, and absorbed by a systematic process. The collective acknowledgement of collective trauma can then become starting point for the societal mobilization of healing mechanisms, through which society is equipped and empowered to draw strength, and finds new pathways to move towards a vision of culture rooted in collective healing.

## Data Availability

Data sharing not applicable to this article as no datasets were generated or analyzed during the current study.

## Footnotes

1 In Timm O´Brien´s “In the Lake of the Woods” (1995), the lake presents as a psychological sphere into which all the imageries like violence and traumata flow and interflow. Lake explores the problem of both personal and collective trauma, c.f. Judith Herman´s “Trauma and Recovery” (1997).

2 Lankshear (1997:21, 24) points out that there are three functions of language which contribute to constructing individual and group identities in cultural process: (1) “language as a precondition of culture and an outcome of cultural process”; (2) “language as medium of culture”; and (3) “language as a broker of cultural process”.
